# Process based modelling of plants–fungus interactions explains fairy ring types and dynamics

**DOI:** 10.1038/s41598-023-46006-1

**Published:** 2023-11-14

**Authors:** Nicole Salvatori, Mauro Moreno, Maurizio Zotti, Annalisa Iuorio, Fabrizio Cartenì, Giuliano Bonanomi, Stefano Mazzoleni, Francesco Giannino

**Affiliations:** 1 Department of Agricultural Sciences, University of Naples Federico II, via Università 100, 80055 Portici, Italy; 2 1DI4A, Department of Agri-Food, Environmental and Animal Sciences, University of Udine, via delle Scienze 206, 33100 Udine, Italy; 3 Department of Life Sciences, University of Trieste, 34127 Trieste, Italy; 4 Faculty of Mathematics, University of Vienna, Oskar-Morgenstern-Platz 1, 1090 Vienna, Austria; 5 Parthenope University of Naples, Department of Engineering, Centro Direzionale-Isola C4, 80143 Naples, Italy

**Keywords:** Ecological modelling, Microbial ecology, Theoretical ecology

## Abstract

Many mushroom-forming fungi can develop circular colonies affecting the vegetation in a phenomenon named fairy rings. Since the nineteenth century, several hypotheses have been proposed to explain how fairy ring fungi form ring-like shapes instead of disks and why they produce negative or positive effects on the surrounding vegetation. In this context, we present a novel process-based mathematical model aimed at reproducing the mycelial spatial configuration of fairy rings and test different literature-supported hypotheses explaining the suppressive and stimulating effects of fungi on plants. Simulations successfully reproduced the shape of fairy rings through the accumulation of fungal self-inhibitory compounds. Moreover, regarding the negative effects of fungi on vegetation, results suggest that fungal-induced soil hydrophobicity is sufficient to reproduce all observed types of fairy rings, while the potential production of phytotoxins is not. In relation to the positive effects of fungi on plants, results show that the release of phytostimulants is needed to reproduce the vegetation patterns associated to some fairy ring types. Model outputs can guide future experiments and field work to corroborate the considered hypotheses and provide more information for further model improvements.

## Introduction

Fairy Rings (FRs) are biological formations caused by fungi. They can be detected by the regular arrangement of sporophores and/or circular bands of changing vegetation that are caused by dense mycelial fronts radially expanding in the soil^1–3^. FRs are formed by a vast number of fungal species, mainly basidiomycetes^4^, both saprotrophic and mycorrhizal^2^. These formations can be found in both natural and anthropized ecosystems such as grasslands, forests, pastures, dunes, managed lawns, gardens and, rarely, in cultivated fields^1,5–9^. FRs size can vary from less than a meter to hundreds of meters in diameter^2^. Previous studies, relating the yearly advancement to the overall diameter of the colony, estimated that fungi can live for hundreds of years in the form of advancing fronts^10–13^. The study of FR fungi is one of the oldest topics in soil ecology, with the first studies dating back to1807^14^. During centuries of investigations, the research effort moved in two directions: (1) understanding the mycelium growth dynamics and spatial configurations in the soil, and (2) studying the change in soil chemical and physical characteristics operated by the fungal mycelium and its effect on the vegetation.

Regarding the reconstruction of mycelial dynamics resulting in FRs spatial patterns, mathematical models were developed to reproduce the centrifugal growth of the mycelium in a homogeneous soil environment assuming the expansion of a ring shape originating from a central starting point^10,15,16^ or, when considering a discontinuous soil environment, reproduced fragmented patterns in which some portions of FRs fronts collapses assuming other shapes such as arcs^17^ and rotors^16^. To the best of our knowledge, the mathematical models explaining FR mycelial dynamics pay little attention to the mechanisms determining why the fungal mycelium disappears inside the colonies resulting in a ring-like shape instead of a disk-like one. Bayliss in 1911, for the first time addressed the question of why those masses of mycelium developed in rings instead of disks. The author proposed that mycelium degeneration in the inner part of the colony could be associated with the accumulation of self-toxic compounds produced by the fungi themselves^7,18–20^ or with the depletion of soil resources^21,22^. Nevertheless, it was observed that the mycelium fronts of *Agaricus tabularis* were unable to recolonize the internal portion of the rings despite resources being restored^1^. Similarly, Dowson, et al.^7^ reported that by inverting soil sods colonized by the *Clitocybe nebularis* in FRs, the mycelium was not able to grow following its natural polarity despite the level of organic matter in soil being restored, suggesting that resource depletion could not be the driving factor of ring formation. Furthermore, the fact that arcs in sloped areas are always found to grow upslope^17,18,23^, strongly supports the self-toxic compound hypothesis as the leaching of a soluble toxic compound downslope is consistent with the degeneration of the downstream portion of the colonies^17,23^. In this context, recently Allegrezza and co-workers^17^ suggested that mycelial degeneration could be caused by the inhibitory role of extracellular self-DNA^24^ creating a parallel between the process observed in fungal colonies with those of tussocks and bushes, where the inner and older parts of plant colony degenerate developing in rings or other regular patterns^25^. Self-DNA has been proven to be a general biological inhibitor^24,26^ and, being also a water-soluble compound, it can be addressed as the putative self-inhibitory compound causing FRs formation as well.

**Figure 1:**
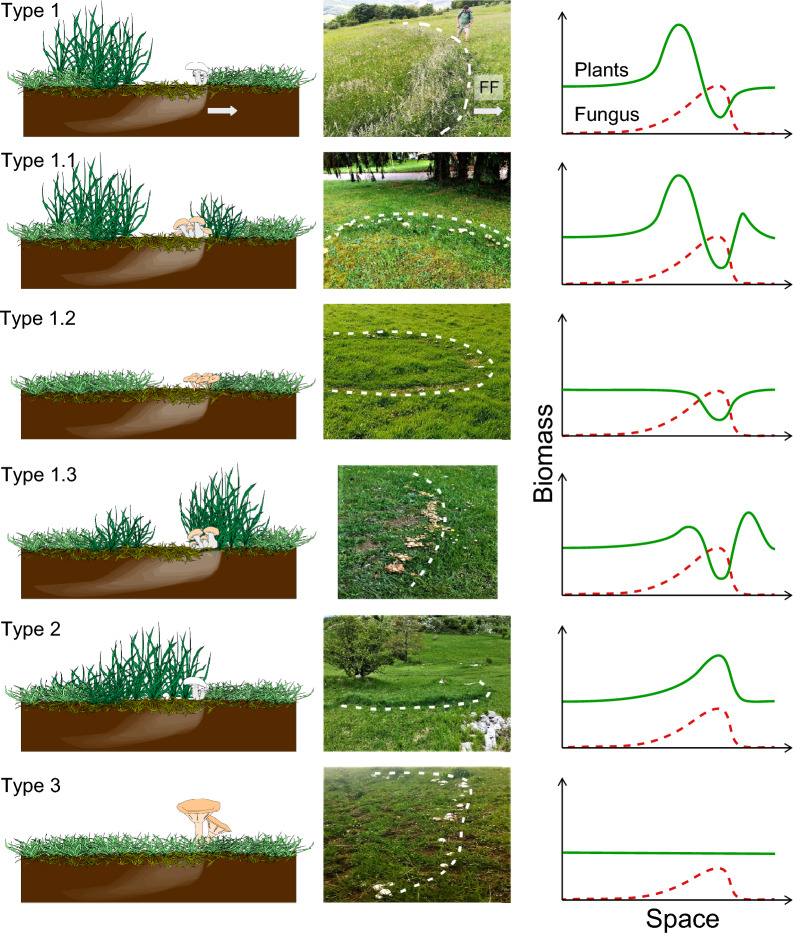
The 5 types of fairy rings observed in nature. On the left, graphical representation of the mycelium distribution in the soil, position of the fungal carpophores, and effect on vegetation; in the middle, dashed lines representing fungal front on real cases pictures of all types; on the right, expected spatial transects of plant and fungal biomass. The two arrows shown for Type 1 figures represent the general direction of expansion for all fairy rings. Pictures from Maurizio Zotti.

Concerning the effects of mycelial mats on vegetation, studies are mainly focused on grasslands where FRs are detected by their different effects on the surrounding vegetation^1,27^ which can be stimulated and/or suppressed by the fungus^28–31^. In many cases, these two opposite effects on plants are both observed on the same ring, either in different moments or, at the same time, in different portions of a FR, e.g. inside or outside the mycelium ring^28,32^. Consequently, FRs have been classified by Shantz & Piemeisel in 1917 into three main types, according to the effect on vegetation and the alternation of dead/stimulated grass cover inside the colony (Fig. 1): *type 1* is characterized by an area of dead vegetation in correspondence with the underground-expanding fungal front followed by a belt of flourishing vegetation^33,34^; *type 2* is characterised by a belt of luxuriant vegetation, without areas of dead vegetation in the proximity^35^; and *type 3* which presents no evident effect on vegetation and is only recognisable by the presence of fungal sporophores^36^. Besides this classification, at least three more types of FRs have been reported, which, according to the classification of Shantz & Piemeisel, can be considered as subtypes of *type 1* since they show both detrimental and stimulating effects on vegetation. These FRs types can be described as (Fig. 1): *type 1.1* presents an additional belt of stimulated vegetation preceding the belt of dead vegetation^6,37^; *type 1.2* where the belt of dead vegetation in correspondence of the fungal front, is not preceded nor followed by any flourishing vegetation belt^27^; and the very rare *type 1.3*, which is similar to type 1.1 but with stronger stimulation of the vegetation belt that precedes the dead vegetation (author’s observation, picture in Fig. 1). The mechanisms driving these different effects on plant cover can be connected to the strategy by which the mycelium colonizes the soil to get resources. When resources are encountered along the path of the fungal mycelium, hyphal branching occurs to monopolize such resources and avoid competition with other fungi. During this process, mycelial mats compartmentalize the soil patch through the release of hydrophobins, enabling fungi to regulate the soil environment and get exclusive access to nutrients from the hydrophobic organic matter. At the same time, some fungi release toxic chemicals and antibiotics to defend the substrate from other species. In summary, the main hypotheses proposed as mechanisms of suppression of vegetation are: (1) *Hydrophobicity*—the reduction of soil permeability to water due to a physical saturation of soil pores with hydrophobic mycelium^32,38,39^; (2) *Phytotoxicity*—the release of phytotoxins like cyanide^40–42^. Several other mechanisms such as immobilisation of nutrients^43^, and direct pathogenic behaviour^18,38,44^ have been proposed, however, these have limited support in the scientific literature. On the other hand, the positive effects of FR fungi on plants have been related mainly to two hypotheses: (1) *Nutrient release*—nutrient enrichment in soil^28,45^; (2) *Phytostimulation*—the direct production by the FR fungus of hormone-like phytostimulants, also known as fairy-chemicals^46^. Although some of the abovementioned mechanisms of plant-fungus interaction have been supported by experimental analyses^32,43,47^, no agreement exists about their relative importance on the morphogenesis of the observed types of FRs.

Even if FRs are convenient formations for the study of the effects of fungi on biological communities^9,48^, the edaphic nature of these fungi makes the study of their mechanisms of action a challenging work. Moreover, the different times of sampling during the vegetative season, the differences in the ecosystems where FRs are found, and the variety of fungal species, make it difficult to experimentally assess a unique explanation for all the reported types of FRs. To overcome the biological and practical limitations for the experimental study of FRs (i.e., slow growth and mycelium detection in soil), we developed a general process-based mathematical model describing the dynamics in space and time of a FR fungus, the plant community and their direct and indirect interactions. In this context, this work aims to:

1. reproduce the formation of FRs taking in account the self-inhibitory role of self-DNA explaining why FRs develop in rings instead of disks;
2. explain the formation of the different types of FRs observed in nature by testing and combining the different abovementioned hypotheses producing negative (Hydrophobicity, Phytotoxicity) and positive (Nutrient release, Phytostimulation) effects on vegetation;
3. provide a list of biometric parameters regarding FRs which can be measured, to validate the model and to independently test the relative effect of each hypothesis.

## Materials and methods

### Model description

**Figure 2:**
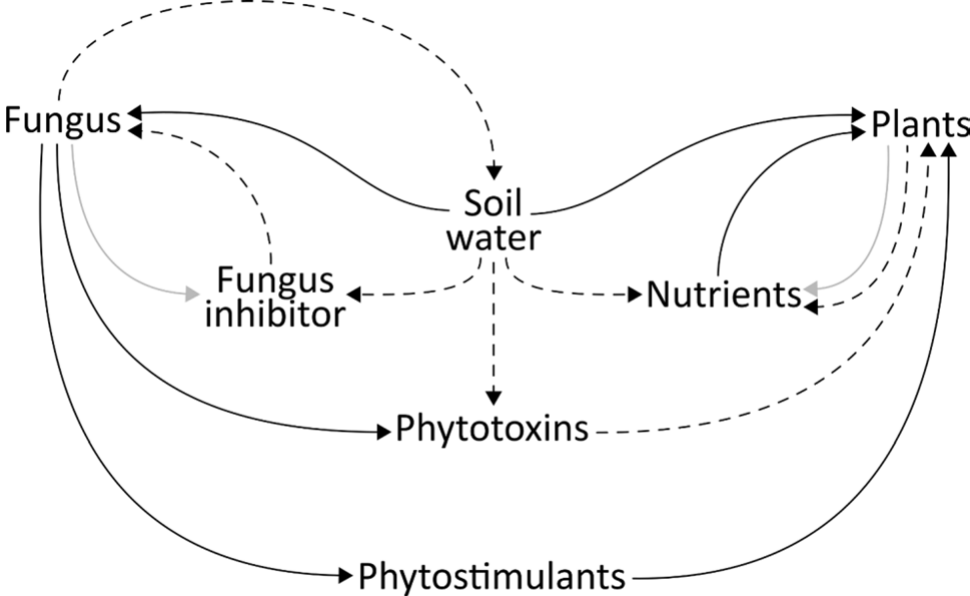
Schematic representation of the modelled elements and their interactions. Positive interactions are represented with solid arrows, dashed lines indicate negative interactions. Grey arrows indicate the release of Fungus inhibitor and Nutrients after the death of Fungus and Plants, respectively.

The mathematical model consists of six partial differential equations describing the formation of FRs and the associated interactions between plants and fungi. Figure 2 shows a schematic diagram of the model with the processes implemented and the interactions occurring among the considered variables. The model is characterised by six state variables: $$F$$ (fungus), $$I$$ (fungal self-inhibitor), $$T$$ (phytotoxins), $$P$$ (plants), $$S$$ (phytostimulants), and $$N$$ (nutrients), which are all calculated as densities (in g dm^−2^). The dynamics in space and time of each state variable are described by one of the six differential equations which make up the model (Eqs. 1–6). In addition to these six state variables, the diagram showed in Fig. 2 presents an additional variable that is fundamental to describe the model: Soil Water which indicates both the water in the soil available for fungus $$(W)$$, and the water in the soil available for plants and for leaching processes ($$\overline{W })$$. Figure 2 also shows the main interactions between the variables. The growth of the FR fungus is modelled as dependent on soil water ($$W)$$ and limited by the accumulation of a fungal inhibitor (*I*). The presence and effect of the fungal inhibitor recalls the hypothesis firstly proposed by Bayliss (1911)^18^, who suggested that fungal colonies develop in rings and not in disks because of the production—by the fungus itself—of a harmful substance hindering the recolonization of the mycelium in previously occupied soil. The inhibitory effect on the fungus is implemented in the model as both a limiting factor on the growth and a cause of extra mortality, this type of implementation follows the work of Cartenì et al. (2012)^25^ concerning ring formation in clonal plants. The fungal inhibitor (*I*) accumulates in the soil after the death of the fungus^24,26^, and then slowly degrades or leaches to lower layers of the soil. These interactions between fungus and its inhibitor determine the fungal ring, thus the characteristic ring shape of FRs.

In addition, the interactions between the other variables of the model are related to the positive and negative effects of FR fungi on plants and specifically refer to the main hypotheses proposed in FRs literature. Consistently with the *“hydrophobicity hypothesis”,* according to which the hydrophobic mycelium mat^49–51^ reduces the infiltration of water inside the soil^1,18,29,32^, the fungus (*F*) reduces the availably of soil water ($$W)$$ for plants. The fraction of soil water available for plants ($$\overline{W }$$) changes dynamically as the concentration of the fungi (*F*) and soil water (*W*) changes (Eq. 7) (Fig. S1). It must be noted that fungal growth (Eq. 1) is directly influenced by the soil water (*W*) and not by the portion of soil water available for plants ($$\overline{W }$$), this is reasonable since fungi, in contrast to plants whose roots are more localized, can capture water from the non-hydrophobic soil situated at the border of the mycelium body^52^. Consistently with the *“phytotoxins hypothesis”*, the model assumes that the fungus produces phytotoxic compounds (*T*), such as cyanide^42,53^, that exert a detrimental effect on plant growth. It is assumed that the fungus produces phytotoxins at a constant rate and are reduced by both degrading at a constant rate and leaching as a linear function of soil water ($$\overline{W }$$).

The model assumes that the fungus produces phytostimulants (*S*)^46,54^ which, once released in the environment, enhance plant growth and degrade at a constant rate (due to volatilization and biological or chemical degradation). Moreover, the death of plants and subsequent decomposition of plant residues determines the enrichment of soil nutrients (*N*), such as nitrogen, which boost the growth of new vegetation. Nutrients can be both absorbed by plants and leached by water. Other existing hypotheses, related to the different effects of FRs on plants, such as the immobilization of nutrients^43^ and the direct pathogenic behaviour^38^, are not included in the model due to their limited support in scientific literature.

Therefore, the growth of the plants’ community depends on the variables related to the main hypotheses described for the positive and negative effects of FR fungi: available soil water $(\overline{W }$), phytotoxins (*T*), phytostimulants (*S*), and nutrients ($N$). Self-inhibition for plants is not modelled as for the fungus, since the self-DNA hypothesis^24^ assumes the inhibitory compounds to be species-specific, whereas in this case we are considering plants at the community level. Considering the spatial and temporal resolutions of the presented model (dm and days respectively), we explicitly model the spatial dynamics of fungi and plants. For the sake of simplicity, no horizontal movement of the other state variables was considered since they represent chemical compounds whose lateral diffusion in the soil can be assumed to be negligible.

The mathematical model is defined by a system of six differential equations:

1 $$\frac{\partial F}{\partial t}={g}_{F}\cdot F\cdot \left(1-{s}_{I}\cdot I\right)\cdot \left(\frac{W}{W+{k}_{W}}\right)-{d}_{F}\cdot {F}^{2}-{s}_{F}\cdot F\cdot I+{D}_{F}{\cdot \nabla }^{2}F$$

2 $$\frac{\partial I}{\partial t}= {c}_{I}\cdot \left({d}_{F}\cdot {F}^{2}+ {s}_{F}\cdot F\cdot I\right)-{k}_{I}\cdot I-{l}_{I}\cdot I\cdot \overline{W }$$

3 $$\frac{\partial T}{\partial t}={c}_{T}\cdot F-{k}_{T}\cdot T-{l}_{T}\cdot T\cdot \overline{W }$$

4 $$\frac{\partial S}{\partial t}={c}_{PS}\cdot F-{k}_{S}\cdot S$$

5 $$\frac{\partial P}{\partial t}={g}_{P}\cdot P\cdot \left[(\overline{W }\cdot \left({q}_{P}+S+N\right)-P\right]-{s}_{T}\cdot T\cdot P+{D}_{P}{\cdot \nabla }^{2}P$$

6 $$\frac{\partial N}{\partial t}={g}_{N}\cdot \left[\left({g}_{P}\cdot {P}^{2}+{s}_{T}\cdot P\cdot T\right)\right]\cdot \overline{W }-{u}_{N}\cdot \left({g}_{P}\cdot \overline{W }\cdot P\cdot N\right)-{l}_{N}\cdot N\cdot \overline{W }$$

where:

7 $$\overline{W }=\left\{\begin{array}{ll}1-{b}^{\left(a\cdot F-W\right)}& if a\cdot F<W\\ 0& if a\cdot F\ge W\end{array}\right.$$

As can be seen in Fig. S1, when the fungus is absent (*F* = 0), $\overline{W }$ grows as a power function with basis *b* (dimensionless) and exponent* W*. The presence of fungus (*F* > 0) leads to a decrease in water infiltration in the soil occupied by the hydrophobic mycelium, reducing both the water available for plants and the magnitude of the processes that depends on soil water (leaching processes).

The description, units, and values of all quantities of the model equations are reported in Table 1. The model has been implemented and numerically solved in MATLAB 2020 (the MathWorks) (Code S1), the timestep is set to one day and all simulations were run for 2000 days (around 5 years and half) and the spatial resolution was set to 0.1 m. All simulations were performed using zero-flux Neumann boundary conditions on a square lattice of 400 × 400. The initial conditions set to zero for all state variables except for *F* which was set to 0.01 only in the central point of the lattice (fungal inoculum), and *P* which was set to 1 on all the domain (grassland vegetation cover). All the plots showing the simulation results have been generated in MATLAB 2020 (the MathWorks).

**Table 1:** List of model state variables and parameters, with relative description, units, and values (initial value for the state variables). The asterisks indicate that these two parameters vary depending on the considered hypothesis. [0.1–0.5] is for the coupled hypothesis (both), 0 for the hydrophobicity hypothesis and [0.9–1.3] for the phytotoxicity hypothesis.

	Symbol	Description	Units	Value
State variables	*F*	Fungus	g dm^−2^	0.01 (initial value)
	*I*	Fungus self-inhibitor	g dm^−2^	0 (initial value)
	*T*	Phytotoxicity	g dm^−2^	0 (initial value)
	*P*	Plant	g dm^−2^	1 (initial value)
	*S*	Phytostimulants	g dm^−2^	0 (initial value)
	*N*	Nutrients	g dm^−2^	0 (initial value)
Parameters	${g}_{F}$	Fungus growth rate	d^−1^	[0.01–0.05]
	${s}_{I}$	Sensitivity of *F* growth to *I*	dm^2^ g^−1^	1
	${k}_{W}$	Michaelis–Menten constant for *W*	–	0.3
	${d}_{F}$	Fungus death rate	dm^2^ d^−1^ g^−1^	0.005
	${s}_{F}$	Sensitivity of *F* death to *I*	dm^2^ d^−1^ g^−1^	2
	${c}_{I}$	Accumulation of *I* due to *F* death	–	0.05
	${k}_{I}$	Degradation rate of *I*	d^−1^	0.001
	${l}_{I}$	Leaching rate of *I*	d^−1^	0.001
	${c}_{T}$	Accumulation rate of *T* due to *F*	d^−1^	0.07
	${k}_{T}$	Degradation rate of *T*	d^−1^	0.07
	${l}_{T}$	Leaching rate of *T*	d^−1^	0.01
	a	Soil water parameter	dm^2^ g^−1^	0/2*
	b	Soil water parameter	–	9
	${g}_{P}$	Plants growth rate	dm^2^ d^−1^ g^−1^	0.9
	${q}_{P}$	Baseline *P* biomass	g dm^−2^	1
	${s}_{T}$	Sensitivity of *P* death to *T*	dm^2^ d^−1^ g^−1^	[0.1–0.5]/0/[0.9–1.3]*
	${c}_{S}$	*S* accumulation rate	d^−1^	0.07
	${k}_{S}$	*S* degradation rate	d^−1^	0.07
	${g}_{N}$	*N* accumulation due to plant death	–	0.08
	${u}_{N}$	*N* plant uptake rate	–	0.3
	${l}_{N}$	*N* leaching rate	d^−1^	0.1
	*W*	Soil water	–	[0.1–2]
	${D}_{F}$	Fungus diffusion rate	dm^2^ d^−1^	0.01
	${D}_{P}$	Plants diffusion rate	dm^2^ d^−1^	0.01

The main set of model simulations were performed to test the “*hydrophobicity hypothesis*” and the “*phytotoxicity hypothesis*” to assess the relative effect of each one on the formation of the different types of FRs and the overall effect if both conditions concur. To do so, the abovementioned processes are turned off alternately through setting to zero the parameters *a* (for hydrophobicity) and ${s}_{T}$ (for phytotoxicity).

### Correlation between model parameters and FR biometry

**Figure 3:**
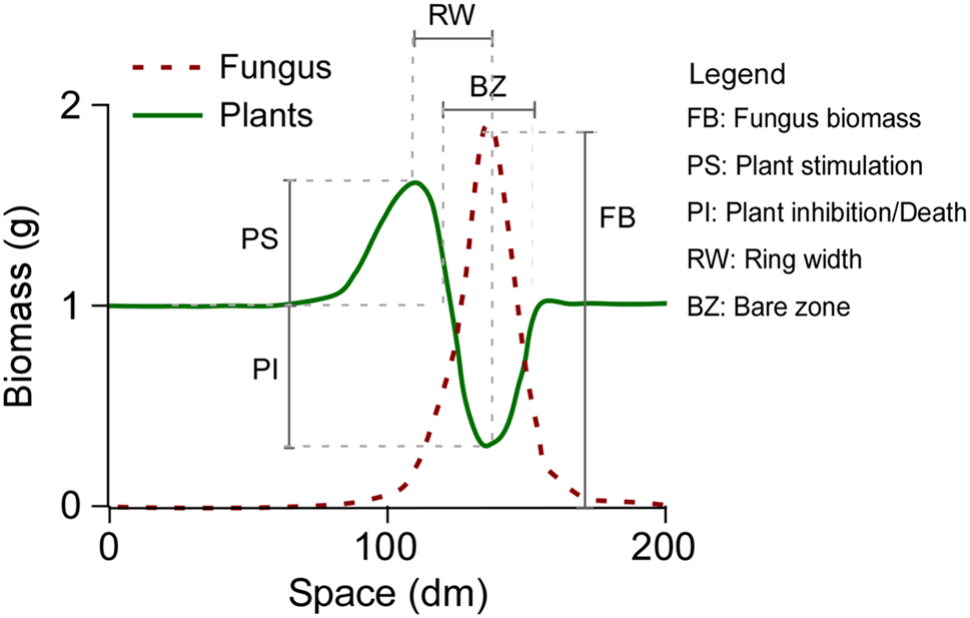
Representation of the biometric parameters and indexes described for fairy rings. Red dashed line represents the growth of the FR fungus. Green continuous line represents the biomass of the plant community (vegetation cover).

To correlate model simulations with measurable quantities, we identified the following biometric parameters (Fig. 3): fungal biomass (FB), calculated as the peak of mycelium biomass; plant stimulation (PS), calculated as the difference between the peak of aboveground biomass in the stimulated band and the steady-state value, i.e. the near unstimulated vegetation; plant inhibition (PI), calculated as the maximum decrease in living plant biomass compared to the steady-state value; the bare zone (BZ), calculated as the width of the belt of plant inhibition; the ring width (RW), calculated as the distance among the fungal and plant biomass peaks. We then changed each model parameter by ± 50% while keeping all the others constant and calculated the Pearson correlation between each parameter and the considered quantities, obtaining positive and negative correlations. Per each quantity and parameter considered, we calculated the fold change (*fc*) for each metric-parameter couple as follows:

8 $$f{c}_{ij}=\frac{\mathrm{max}\left({X}_{ij}\right)-\mathrm{min}\left({X}_{ij}\right)}{\mathrm{min}\left({X}_{ij}\right)}$$

where ${X}_{i}$ is the *i*th quantity (FB, PS, PI, BZ, RW), *j* is the *j*th model parameter and *max* $\left({X}_{ij}\right)$ and $min({X}_{ij})$ are the maximum and minimum values obtained per quantity ${X}_{i}$ by changing the *j*th parameter by ± 50%. This quantity is always positive.

We finally constructed a heatmap showing the correlation among the parameters and these quantities (by indicating only the sign of this correlation) and the intensity of this variation (the fold change). To allow a clear graphical representation, the heatmap is normalized per column, so that the changes in colour intensity are relevant only if considered column by column.

## Results

**Figure 4:**
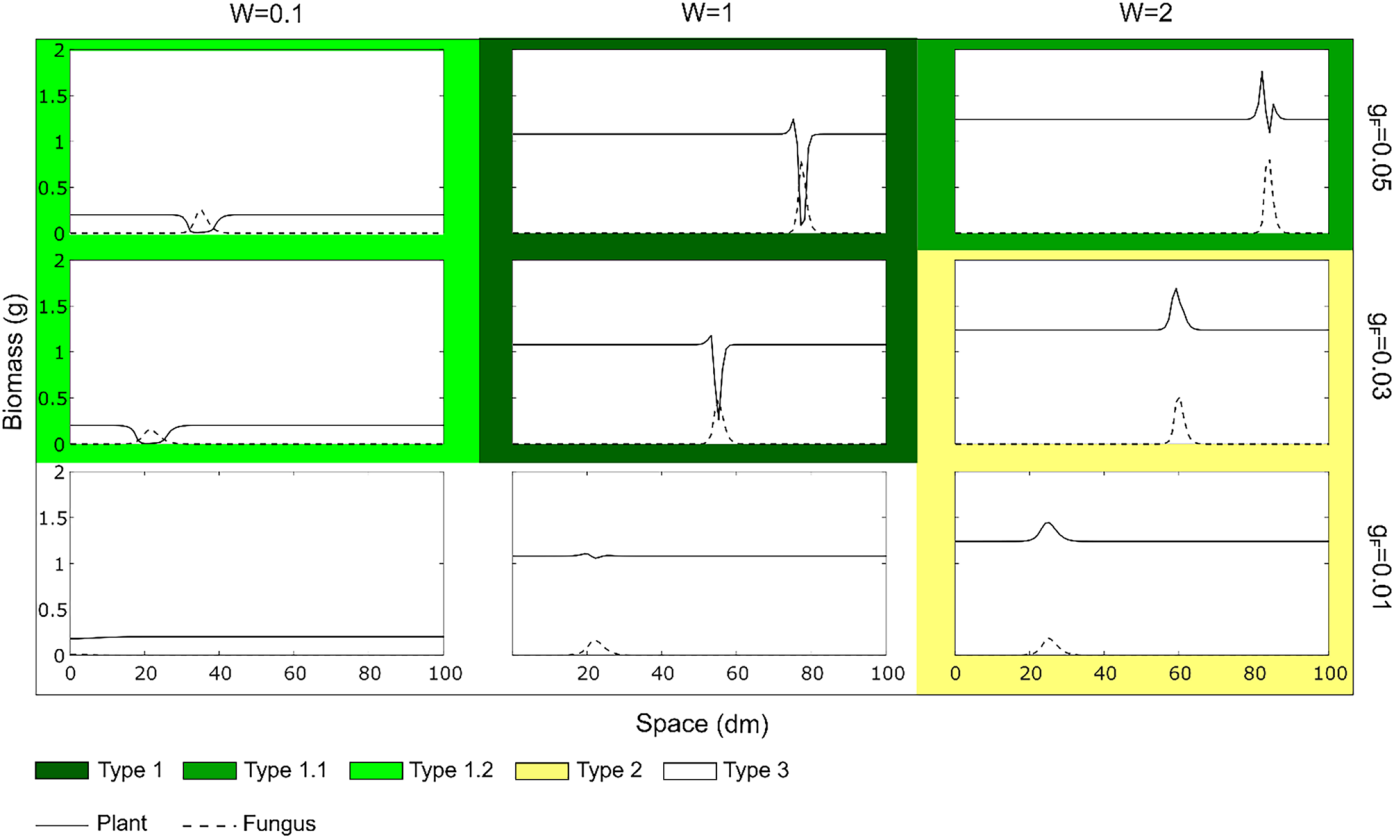
Panel showing the results of the modelled plants and fungi biomasses (transects in space) for the hydrophobicity hypothesis. The parameters related to the levels of soil water (W) and the growth rate of the fungus (${\mathrm{g}}_{\mathrm{F}}$) have been varied. Each one of the five types of FRs are represented with different colours. Intermediate types of FRs are shown with two colours. Other parameter values are reported in Table 1.

The first two equations (Fungal biomass *F* and Fungal inhibitor *I*) allow to represent the formation of a FR pattern. The simulations show that the ring-like shape only emerges in the presence of a self-inhibitory effect (${s}_{I}$ > 0); an increase in the impact of the inhibitor on the fungi, both decreases the equilibrium value and the fungal biomass peak, i.e., a lower biomass density of the fungus at the front and in the inner part of the ring (Fig. S2). Then the different intensities of plant-fungus interaction are qualitatively represented by the model which can describe the different types of FRs (Fig. 1). Here we report the results obtained for the two hypotheses and the combination of them. In Fig. 4 the results of the model in the context of the *hydrophobicity hypothesis* show how the model outcomes (plant and fungal biomass in a transect starting from the centre of the ring after 5 years) change for variations in the fungal growth rate parameter (${g}_{F}$) and in soil water (*W*). Other environmental conditions and differences of fungal species were not modelled explicitly but, in term of effects on fungal biomass, they are associable with variations of the fungal growth rate ${(g}_{F})$. In this case, the simulations indicate that by increasing soil water, both plant and fungal peaks increase, while the bare zone of the FR and the plant inhibition decrease. Instead, by increasing the fungal growth rate, it can be noticed both an increase in the plants and fungal biomass at the peaks and an increase in fungal front velocity. Figure 4 shows that the *hydrophobicity hypothesis* is sufficient to reproduce five types of FRs. However, by performing a parameter exploration, we found that the same model can also simulate *type 1.3*, characterised by a belt of luxuriant vegetation (a plant biomass peak) before the passage of the fungus which is higher than the one after. *Type 1.3* was represented by setting the parameters related to the accumulation and degradation of phytostimulants to ${c}_{S}$= 0.8 and ${k}_{S}$= 1, to the accumulation of nutrients to ${g}_{N}=0.2$ and with W = 2 and ${g}_{F}$ = 0.05 (Fig. S3). The parameters related to phytostimulation were set in a way that describes a fungus able to release many volatile compounds (${c}_{S}$) with a fast rate of volatilization (${k}_{S}$). The spatial and temporal evolution of the system is shown in Supplementary Video 1, representing the plant and fungal biomasses using the parameter set *W* = 1 and *g*_*F*_ = 0.05. After the first time steps when the fungal peak forms, the simulation shows that the fungal front behaves as a travelling wave, i.e. a front with constant shape that only moves in space.

**Figure 5:**
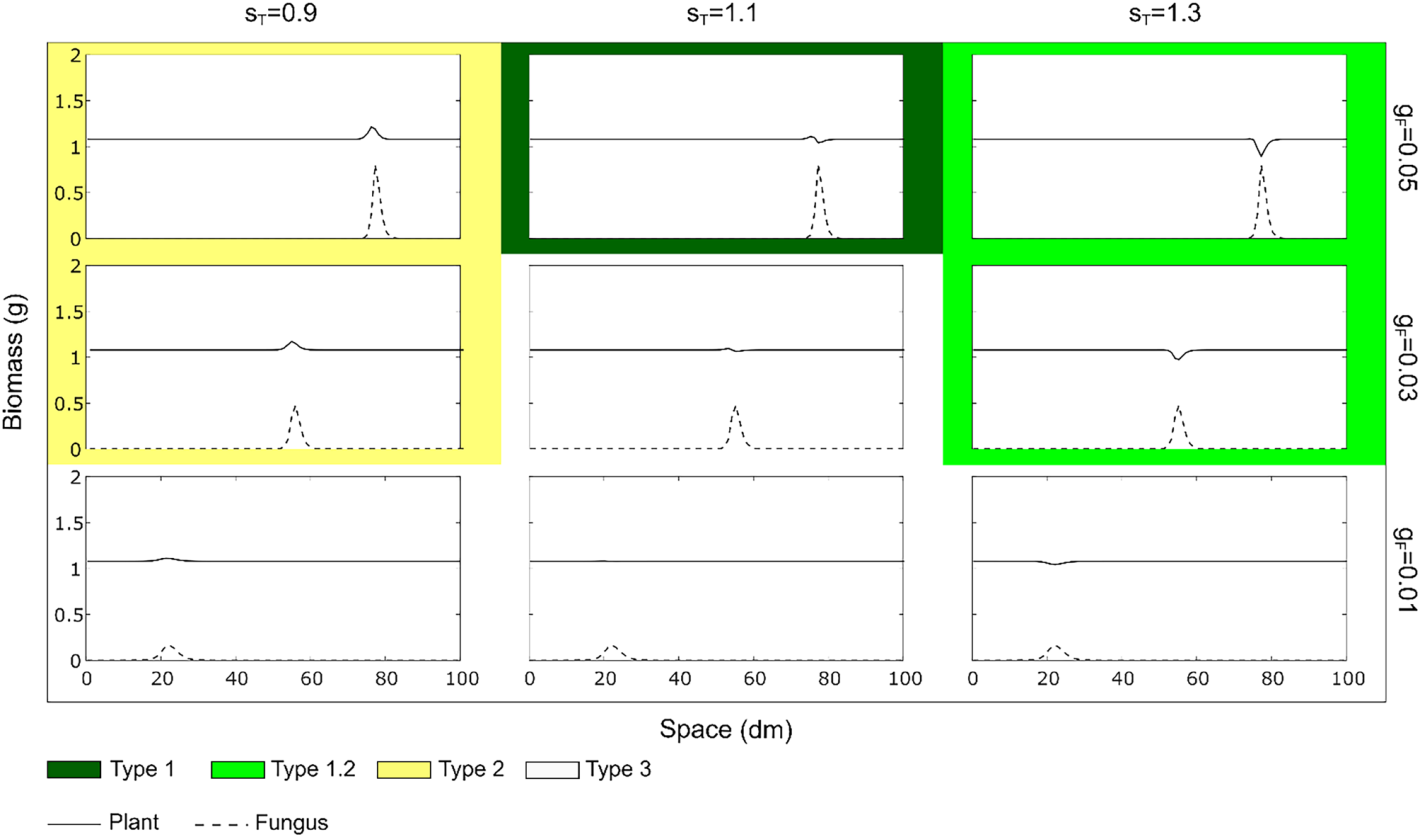
Panel showing the results of the modelled plants and fungi biomasses (transects in space) for the phytotoxicity hypothesis. The parameters related to the sensitivity of the plant to phytotoxicity (${\mathrm{s}}_{\mathrm{T}}$) and the growth rate of the fungus (${\mathrm{g}}_{\mathrm{F}}$) have been varied. Each one of the five types of FRs are represented with different colours. Intermediate types of FRs are shown with two colours. Other parameter values are reported in Table 1.

Then, Fig. 5 shows the results of the model in the context of the *phytotoxicity hypothesis*. In this case, the panel shows how the model outcomes change with different values of the fungal growth rate (${g}_{F}$) and the parameter ${s}_{T}$ which is the sensitivity of the plant to phytotoxins released by the fungus. The simulations show a certain level of similarity with the previous hypothesis in terms of changes in ${g}_{F}$. Instead, changes in ${s}_{T}$ enables the transition from *type 2* to *type 1.2* (plant stimulation to plant inhibition). Due to the nature of this model, we were not able to reproduce the *type 1.1* (plant stimulation to inhibition and followed by higher stimulation) and *type 1.3*, independently from the parameters chosen.

**Figure 6:**
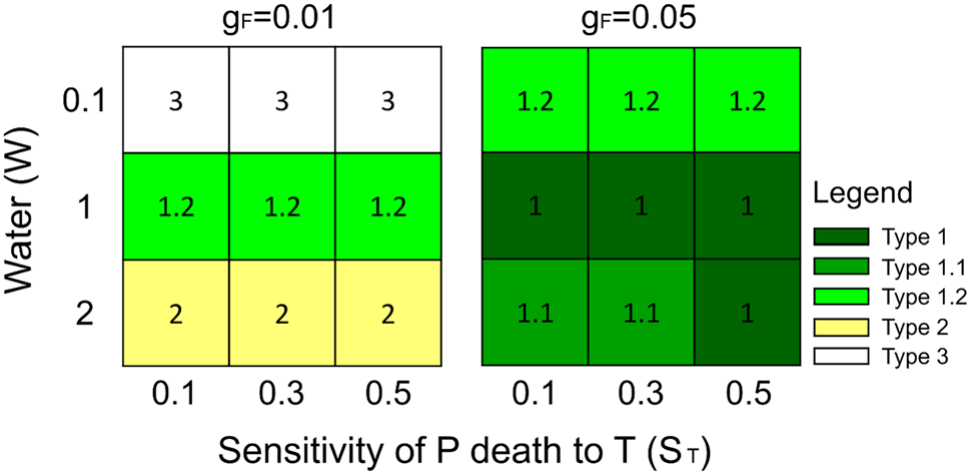
Results of modelled plants and fungi biomass with combined effects of both hydrophobicity and phytotoxicity. All the five types of fairy rings are found according to the different combinations of parameters related to the sensitivity of the plant to phytotoxicity (${\mathrm{s}}_{\mathrm{T}}$), soil water (W) and fungus growth rate (g_F_). Details in the model results can be found in Figs. S4 to S7. Other parameter values are reported in Table 1.

Instead, Fig. 6 illustrates the results of the model combining the two hypotheses and changing ${g}_{F}$, ${s}_{T}$ and *W*. As shown in Fig. 6 this combined version can simulate 5 different types of FRs. Moreover, as in the case where only hydrophobicity was considered, also in this case it is possible to simulate *type 1.3* by setting the parameters related to the accumulation and degradation of phytostimulants to ${c}_{S}$= 0.8 and ${k}_{S}$= 1, and with W = 2 and ${g}_{F}$=0.05 (Fig. S4). Figures S5 and S6 show the panels with fungal and plants biomass as in Figs. 3 and 4 for this combined version of the model. Finally, the results show the relationship between the model parameters (Table 1) and the biometric indicators of the FR (FB, PS, PI, RW and BZ as described in Material and Methods, Fig. 3), highlighting the effect of each parameter on the simulation outcomes. In particular: (1) the fungal biomass (FB) depends on the growth rate of the fungus, its sensitivity to the self-inhibitor, and on the accumulation of the self-inhibitor (${g}_{F}$, ${s}_{F}$ and *c*_*I*_ respectively); (2) the plant stimulation (PS) is positively correlated to the water input (*W*), the phytostimulants production and degradation (*c*_*S*_ and *k*_*S*_), and with phytotoxins degradation (${k}_{T}$); (3) the plant inhibition (PI) is positively correlated with fungal growth rate (${g}_{F}$), water availability (parameters *W*, *a* and *b*), and finally, it is negatively correlated with plant nutrient uptake ${(u}_{N}$); (4) the ring width (RW) is negatively associated with phytotoxins degradation ${(k}_{T}$) and it is positively correlated with phytostimulants degradation (${k}_{S}$); finally, (5) the bare zone (BZ) is negatively correlated with soil water availability ($b$ and $W$) and with the growth rate of the plant community (${g}_{P}$) and positively correlated with the degradation of phytostimulants (${k}_{S}$) and uptake of nutrients (${u}_{N}$). Figure S7 shows the same heatmap for the two separate hypotheses, with the general trend that remains similar to the combined hypotheses. Furthermore, in the hydrophobicity model, the effect of the available water on the biometric quantities is further exacerbated and this reduces the effects of the parameters related to plant dynamics on the biometric quantities, whereas in the phytotoxicity model the parameters related to the phytotoxins and plants and phytostimulants dynamics play a major role.

## Discussion

In the past, very few mathematical models have been developed to reproduce FRs dynamics. These works analysed the spatial development and shapes of fungal colonies (e.g. rings or arcs)^16^ and mainly focused on fungal expansion in the soil^10,55^, without considering why fungal colonies forming FRs degenerate in the central portion. Additionally, the understanding of plants–fungus interactions in FRs is a key point that should not be disregarded since the phenomenon taking place in the soil is indirectly visible through its effects on the aboveground vegetation. To the best of our knowledge, this is the first attempt to model and mathematically analyse both the self-inhibition in fungi and the plants–fungus interactions in FRs.

### Ring development in FRs

Presented model simulations regarding mycelial growth dynamics in the soil (Fig. S2) clearly show that the addition of a process of self-inhibition is sufficient to explain the emergence of a ring-like shape. Following previous modelling work on the development of ring structures in clonal plants^25^, and other experimental findings on the inhibitory role of extracellular self-DNA in plant–soil negative feedback^24^, and in other organisms, including fungi^26^, we propose that fungal self-inhibition, via the accumulation of extracellular DNA of the fungus itself in the soil, is the fundamental process explaining the formation of the FR pattern. This answers the unsolved question posed by Bayliss in 1911^18^ on how the mycelia of FR fungi develop in the shape of rings instead of disks. According to the proposed model, as the fungus grows and moves away from the area of initial occurrence, the turnover of its mycelium in the central part causes the release of the fungus self-DNA that builds up in the soil creating local conditions of species-specific negative feedback. The model also considers the effect of water on DNA, as it is a water-soluble molecule and thus can be washed out and moved deeper in the soil by flooding conditions or precipitation^56^. DNA movement in the soil by water is also proposed to explain why FRs differ in shape according to site geomorphology, as they usually develop in rings in flat conditions while in sloped areas they form upward migrating arcs^17,23^.

### Interaction of FR fungi with vegetation

**Figure 7:**
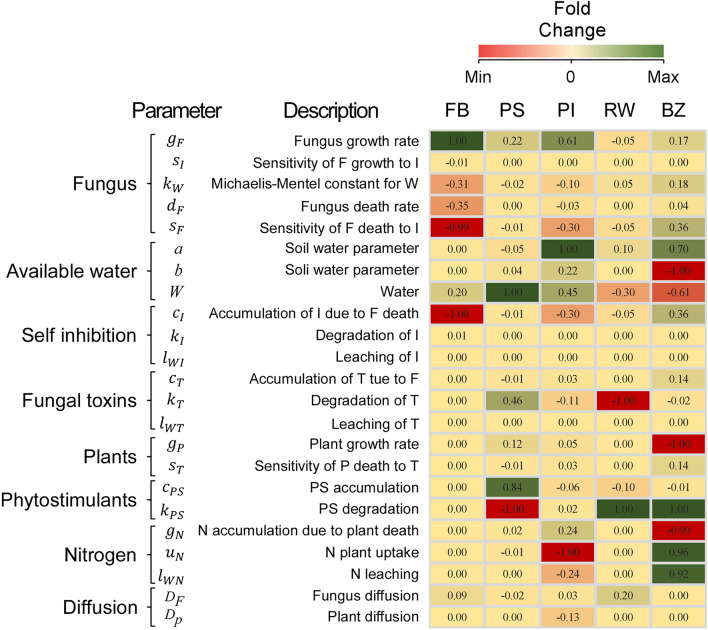
Heatmap showing the correlation (positive or negative) and fold-change (numeric values) between model parameters and FR biometrics for the combined hypotheses. The intensity of each colour is normalized per column. Abbreviations refers to FR biometrics; FB: Fungus biomass; PS: Plant stimulation; PI: Plant inhibition/death; RW: Ring width; BZ: Bare zone. Explanation of indicators in Fig. 3.

To explain the diversity of FRs types and the associated vegetation patterns we developed a general process-based model that considers both stimulating and detrimental effects of fungi on vegetation. Regarding the positive effects on vegetation, previous works scrutinized the role of nutrients released after the passage of FR fungi, showing a poor link between the nutrients released by the fungi (*Marasmius oreades* and *Agaricus arvensis)* and plant growth^28,32^. However, it remains obvious that the decomposition of plant materials creates accumulation of organic matter and nutrients in the soil, and this process is enhanced in such cases where the FR induces mortality of the plant cover (FR *types 1, 1.1, 1.2 and 1.3*). On the other hand, the role of phytostimulants as drivers of plant stimulation has been studied in the FR forming fungus *Lepista sordida*, where the compounds known as “fairy chemicals”, acting similarly to auxin-like hormones, induced incremented growth in different plant species^57,58^. Further support for the production of phytostimulants comes from a more recent work^47^ where it is reported that the fungus *Floccularia luteovirens* can enhance the growth of grasses seedlings releasing a mixture of phytostimulating VOCs (Volatile Organic Compounds). In this context, our modelling results suggest that the production of phytostimulants might play a more relevant role than previously thought, since only this process allows the stimulation of vegetation ahead of the fungal front where no mortality of plants has already occurred and thus no new nutrients have been released in the soil (as in FR *types 1.1* and *1.3*). In conclusion, we assume that both processes of phytostimulation by the fungus and nutrient release by plant litter decay are relevant in FRs formation and dynamics although with different spatial distributions in relation to the fungal front (Fig. 7).

Regarding the negative effects on vegetation, previous studies reported on the role of fungal hydrophobins, that are proteins typically secreted by filamentous fungi^50^. These are known to be produced for defence and sporophore production purposes^51^, and also to provide higher affinity to hydrophobic substrates and soil organic matter in environments where competition for water is high, such as in grasslands^59^. The induction of hydrophobicity by FR fungi is supported by the observation that treating managed grasslands and turfs with wetting agents, helps the vegetation recover from damages caused by FRs^60^. Regarding the production of phytotoxins by FR fungi, several works reported a marked increase in Cyanuric compounds surrounding the mycelium of *M. oreades*^38,40,53^.

Simulation results including all positive and both negative processes (induction of hydrophobicity and production of phytotoxins), were able to reproduce all types of FRs observed in nature (Fig. 6 and S4). However, to evaluate the relative importance of the two negative processes on the suppression of vegetation, we tested them separately. Results show that the model including hydrophobicity as the only negative effect can reproduce all types of FRs (Fig. 4 and S3) while the model without hydrophobicity and only including fungal phytotoxicity, cannot reproduce *types 1.1*, and *1.3* (Fig. 5).

**Figure 8:**
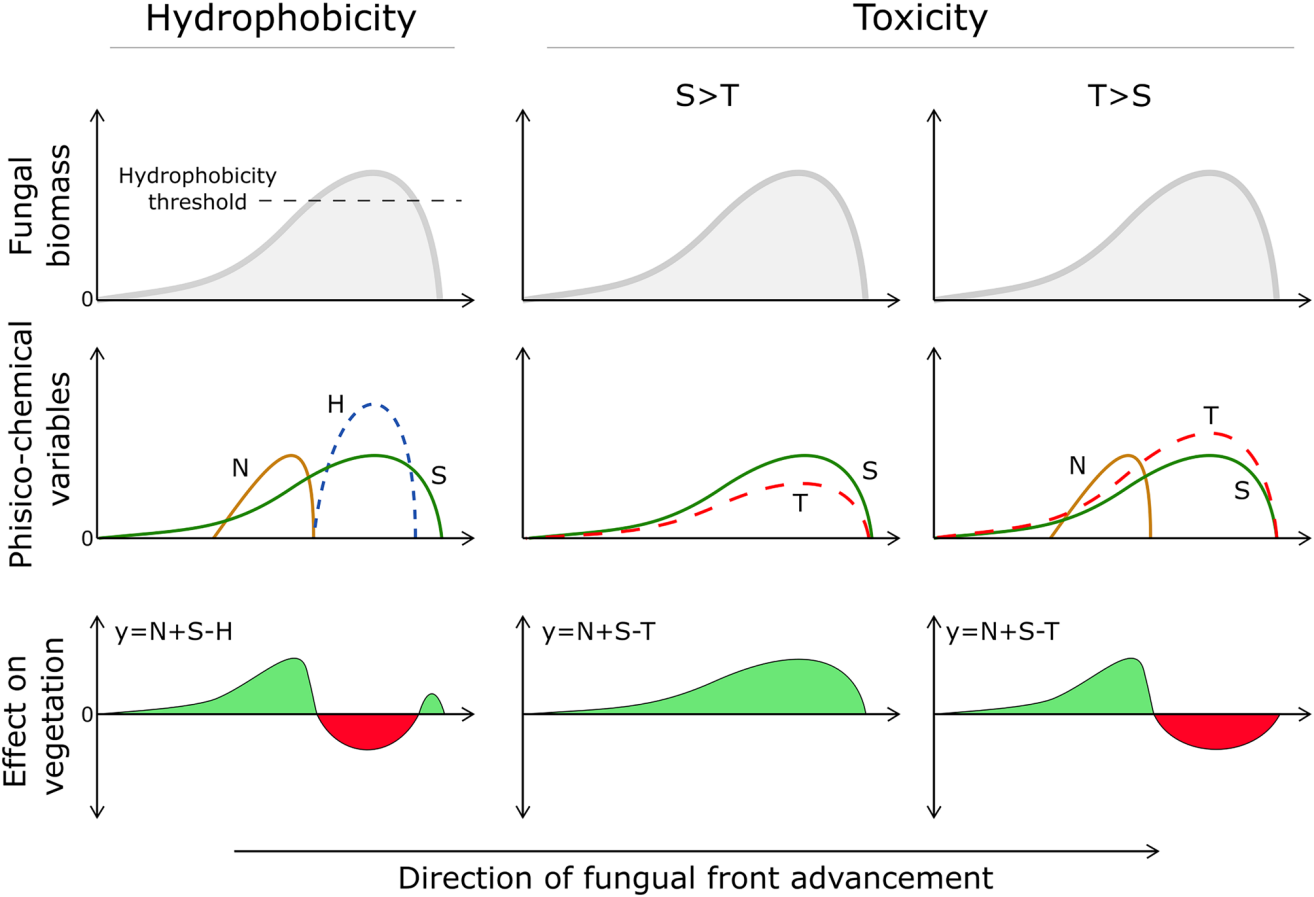
Example of the different effects of FR fungi on vegetation, considering either the Hydrophobicity or the Phytotoxicity hypothesis. N: Nutrients derived by plants decay; S: Phytostimulants released by the fungus; H: Hydrophobicity produced by the fungal biomass above a threshold level; T: Phytotoxic compounds released by the fungus. Bottom row represents the net effect on vegetation of the combination of positive (N, S) and negative (H, T) conditions.

Figure 8 shows a schematic representation of the fungal biomass distribution in a FR transect, with the associated phisico-chemical variables: nutrients (N), hydrophobicity (H), phytostimulants (S), and phytotoxic compounds (T). In particular, N spatial distribution follows the belt in which dead vegetation is decomposing; H occurs only when the mycelium mat density is high (Fig. S1); S and T are proportional to fungal biomass^60,61^, being produced by active metabolism. Therefore, considering the spatial occurrence of these variables, examples of their combinations and net effects on the vegetation are reported in the bottom row of Fig. 8. The first column shows a case where only hydrophobicity is included as negative effect on plants, with the appearance of two areas of stimulated vegetation, both ahead and behind the fungal front (*type 1.1*). Instead, the second and third columns report two cases where only toxicity is included as negative effect. If stimulation overcome phytotoxicity (S > T), the ring is formed only by flourishing vegetation (*type 2*) matching the below ground mycelium density. Otherwise, if phytotoxicity is higher then phytostimulation (T > S), the ring is formed by a bare-zone followed by a stimulated vegetation belt produced by the increased nutrient release (*type 1*).

### Biological and environmental factors defining FRs types

The effect of the fungal mats of FRs on vegetation is mostly due to the ability of the fungus to create hydrophobic soil conditions, which is related to the fungal mycelial density. Accordingly, observations of *Agaricus tabularis* and *Calvatia cyathiformis* mycelial mats, forming respectively *type 1* and *type 2* FRs, reported higher mycelial densities for the first compared to the second^1^. It follows that the fungal species and its colonization strategy modulates the effect on the plant community. However, this may also vary within the same species, e.g., *M. oreades*, mostly described as forming *type 1* FR, has been reported to form also *type 2* in different environmental conditions^1,4,32,37^ and, sometimes, *type 3* as well^61^ with no evident effect on vegetation. Different effects on vegetation by the same fungal species have been observed in contiguous pastures under different management practices in South East England^6^. Similarly, *Calocybe gambosa* (Fig. 1) and *Agaricus campestris* were both described to form *type 1* and *type 2* patterns in different environments and to shift from *type 2* to *type 1* during the same vegetative season^9,33,62^. It has been proposed that a fungal species may produce *type 1* in its optimal environmental conditions and *type 3* at the borders of its ecological niche. This has been observed at different altitudes in the Italian Apennine^17^ and it is consistent with the model results showing that *type 3* emerges at lower mycelial density (Figs. 4, 5, 6). The fungal phenology and the seasonal patterns of precipitation can explain observed differences in FR characteristics. For example, *C. gambosa*, developing in late spring, initially forms *type 2* patterns, later switching to *type 1* when the season becomes drier (personal observation). In our simulations (Fig. 4) *type 1.2* (only dead vegetation without stimulation), *type 1* (both stimulation and suppression), *type 2* (only stimulation) are produced at low, medium and high water levels, respectively. This is consistent with field observations in Mediterranean environments, with dry and warm summers, combined with the presence of hydrophobic mycelium, mostly leading to the emergence of *types 1*, *1.1* or *1.2* FRs with evident decrease of vegetation biomass. In spring, however, when abundant precipitations compensate the effect of the hydrophobic mycelium, ephemeral formations of *type 2* FRs can be observed as transient forms.

### Conclusions and research needs

The presented model was capable to reproduce all known FRs types, consistently with the simple and logical assumptions of (1) self-inhibition of the fungus as driver of ring formation and, (2) plants–fungus interactions as drivers of the emergence of different vegetation patterns. The integration of different mechanisms producing both positive (phytostimulation and nutrient accumulation) and negative (phytotoxicity and hydrophobicity) effects allowed the dynamic simulation of FR structures.

In relation to the formation of ring patterns, a subject of major interest is the investigation of the accumulation of fungal DNA and its role as the self-inhibitory compound in the central FR area.

Previous work analysed the spatial distribution of FRs at a biogeographic scale relating their occurrence to environmental and geomorphological variables^17,23^. Further investigations should be done to analyse the geographical distribution of different FRs types. Moreover, monitoring the FR state of development during the vegetative season will deepen the understanding of changing FR types in association with environmental conditions and vegetation phenology.

Finally, field surveys at local scale should include a focus on the biometric parameters corresponding to different FR types, to evaluate their association to local environmental conditions, different fungal species and their metabolic activity.

### Supplementary Information

Supplementary Video 1.

Supplementary Information 1.

Supplementary Information 2.

## Data Availability

Model code is available as supplementary material (Code S1).
