# Supplementary material for: Process based modelling of plants–fungus interactions explains fairy ring types and dynamics
Source: Sci Rep. 2023 Nov 14;13:19918. doi: 10.1038/s41598-023-46006-1 (PMC10646123; doi:10.1038/s41598-023-46006-1)
Supplement: Supplementary file 2 — Supplementary Information 1. [file 41598_2023_46006_MOESM2_ESM.docx]

Process based modelling of plants-fungus interactions explains fairy ring types and dynamics

**Authors:** Nicole Salvatori^§1,2,3^, Mauro Moreno^§,1^, Maurizio Zotti^*1^ Annalisa Iuorio^4^, Fabrizio Cartenì^1^, Giuliano Bonanomi^1^, Stefano Mazzoleni^1^, Francesco Giannino^1^

**Authors Affiliation**:

^1^ Department of Agricultural Sciences, University of Naples Federico II, via Università 100, 80055 Portici, Italy.

^2^ 1DI4A, Department of Agri-Food, Environmental and Animal Sciences, University of Udine, via delle Scienze 206, 33100 Udine, Italy.

^3^ Department of Life Sciences, University of Trieste, 34127, Trieste, Italy.

^4^ University of Vienna, Faculty of Mathematics, Oskar-Morgenstern-Platz 1, Vienna, 1090, Austria

^§^These authors equally contributed to the work.

^*^ Corresponding author:
Department of Agricultural Sciences

University of Naples Federico II

via Università 100, 80055 Portici, Italy.

E-mail: [ma](mailto:nicole.salvatori@phd.units.it)urizio.zotti@unina.it

Telephone: +39 334 3326848

*
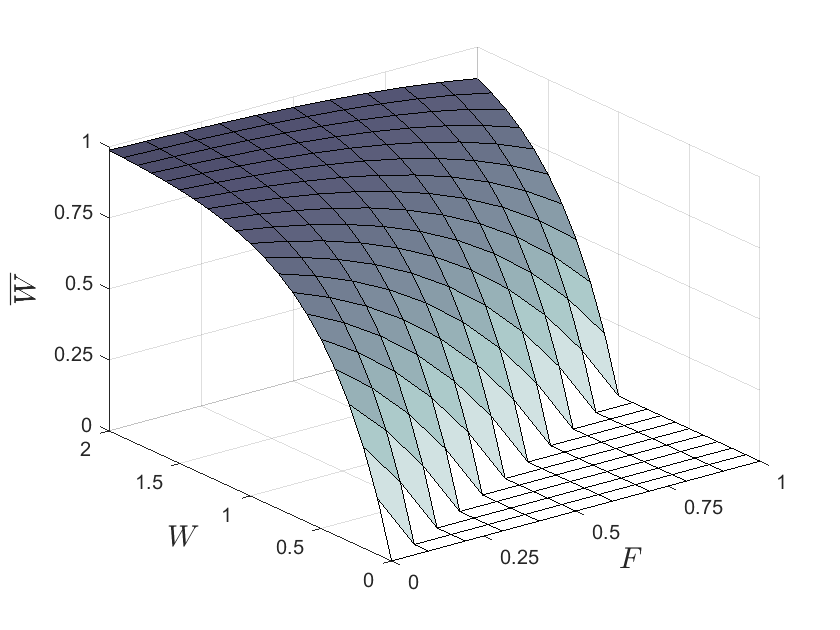
*

***Figure S1.*** *The relation between the fungus and the soil water as described by equation 7.*

*
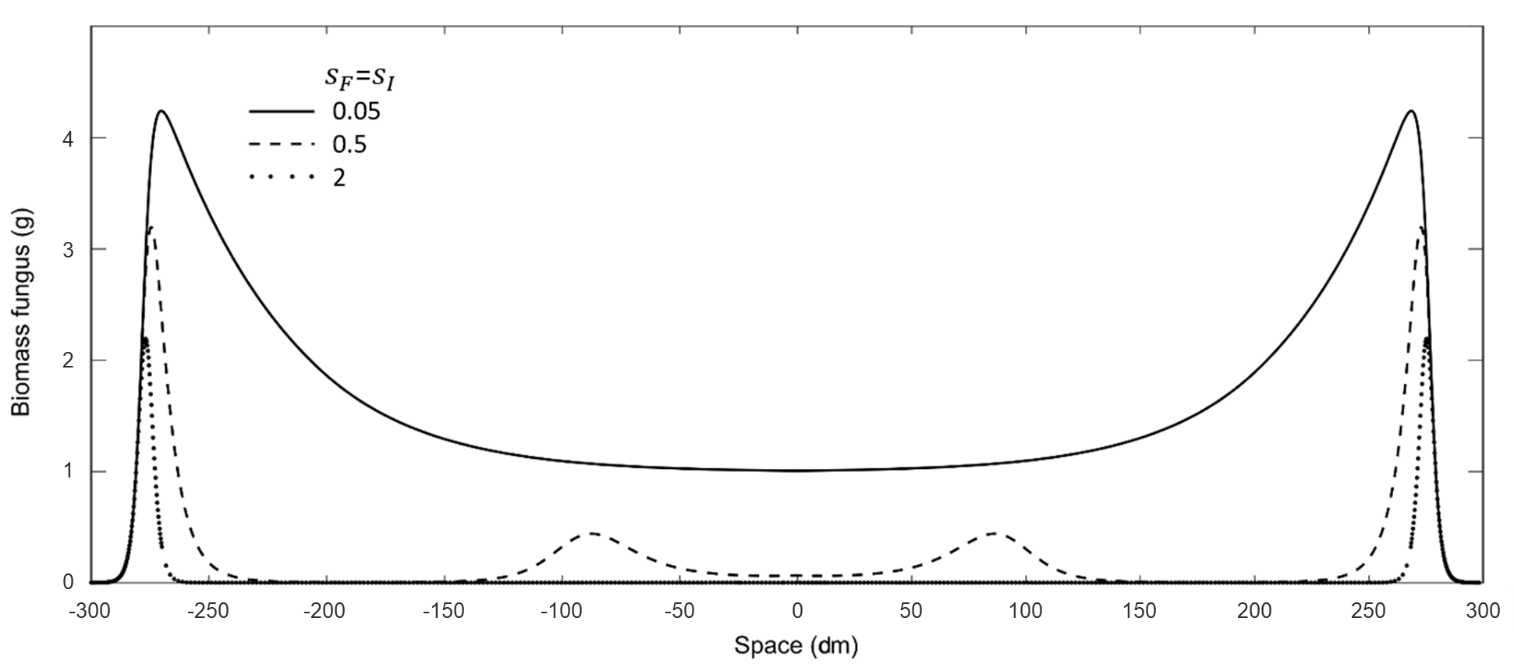
*

***Figure S2****. Fairy ring formation due to different self-inhibitory levels. The curve is the solution of the partial differential equations 1 and 2 (F and I) after 4000 time steps. Different levels of the fungus self-inhibition were tested changing the parameters s_F_ and s_I_ at the same time. Other parameter values are reported in Table 1.*

*
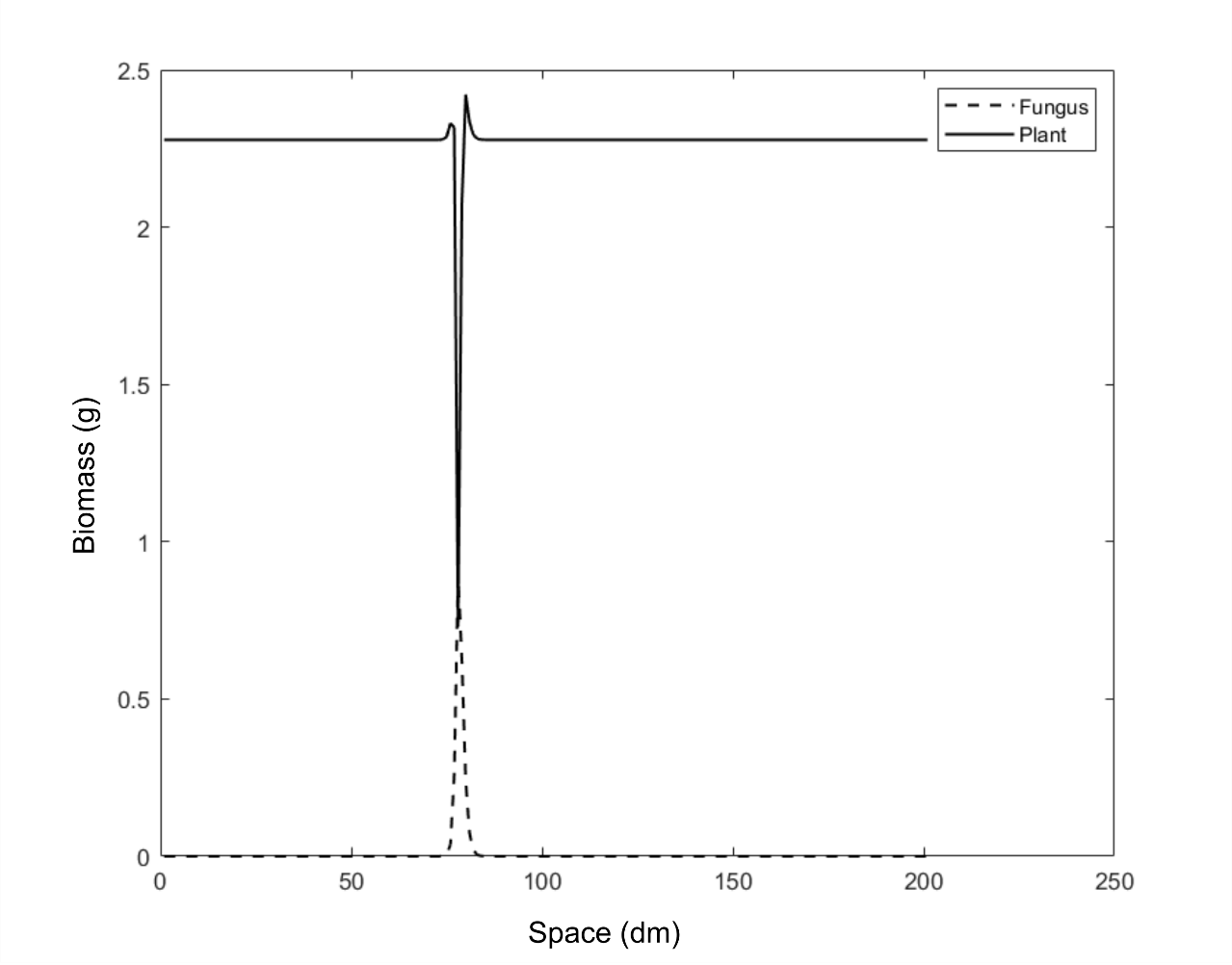
*

***Figure S3.*** *Result of simulation for the hydrophobicity hypothesis with* $c_{S}$*= 0.8,* $k_{S}$*= 1,* $g_{N}=0.2$*, W=2 and* $g_{F}$*=0.05. The resulting FR has been identified as type 1.3.*

***
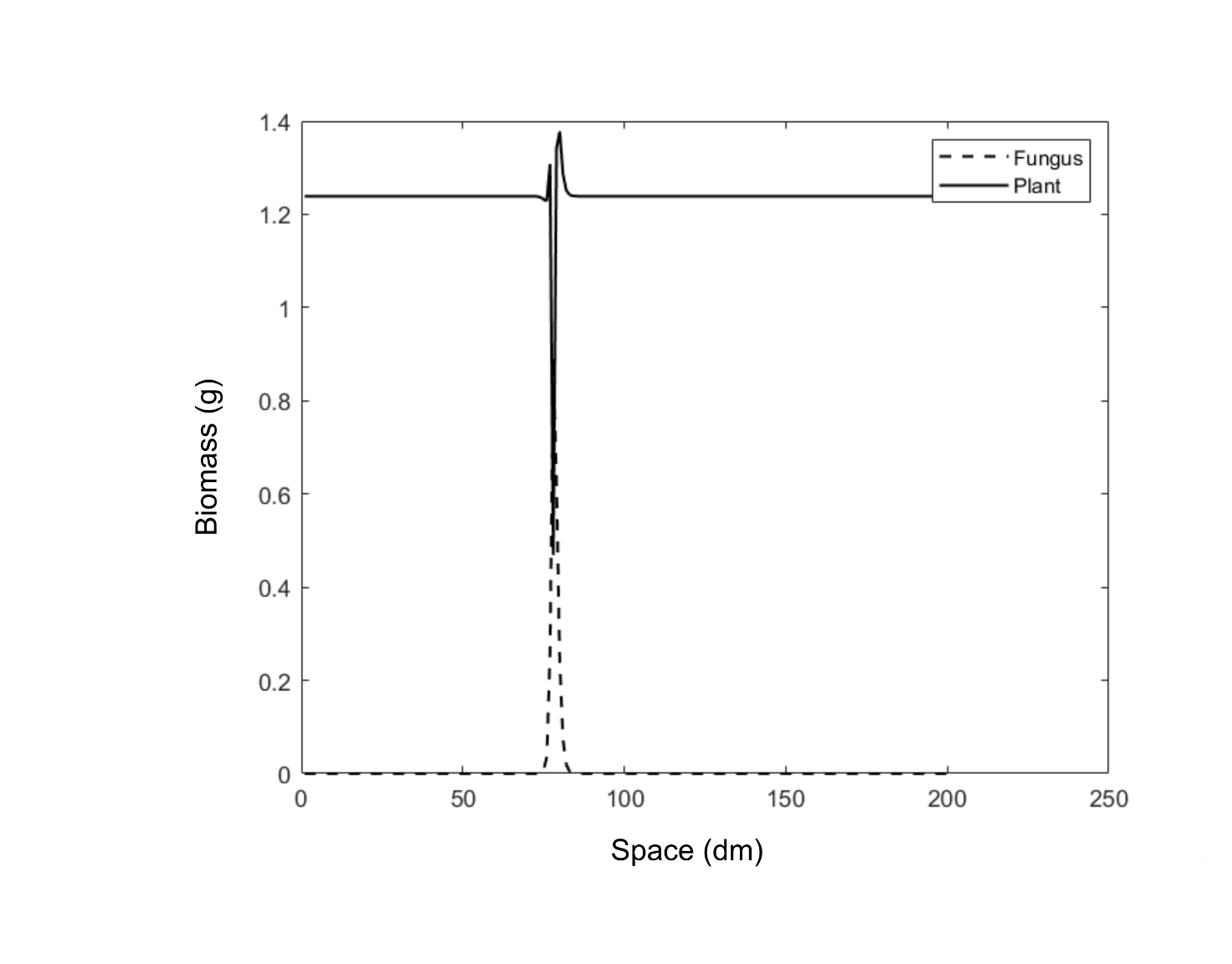
***

***Figure S4.*** *Result of simulation for the combined hypotheses with* $c_{S}$*= 0.8 and* $k_{S}$*= 1, and with W=2 and* $g_{F}$*=0.05. The resulting FR has been identified as type 1.3.*

***
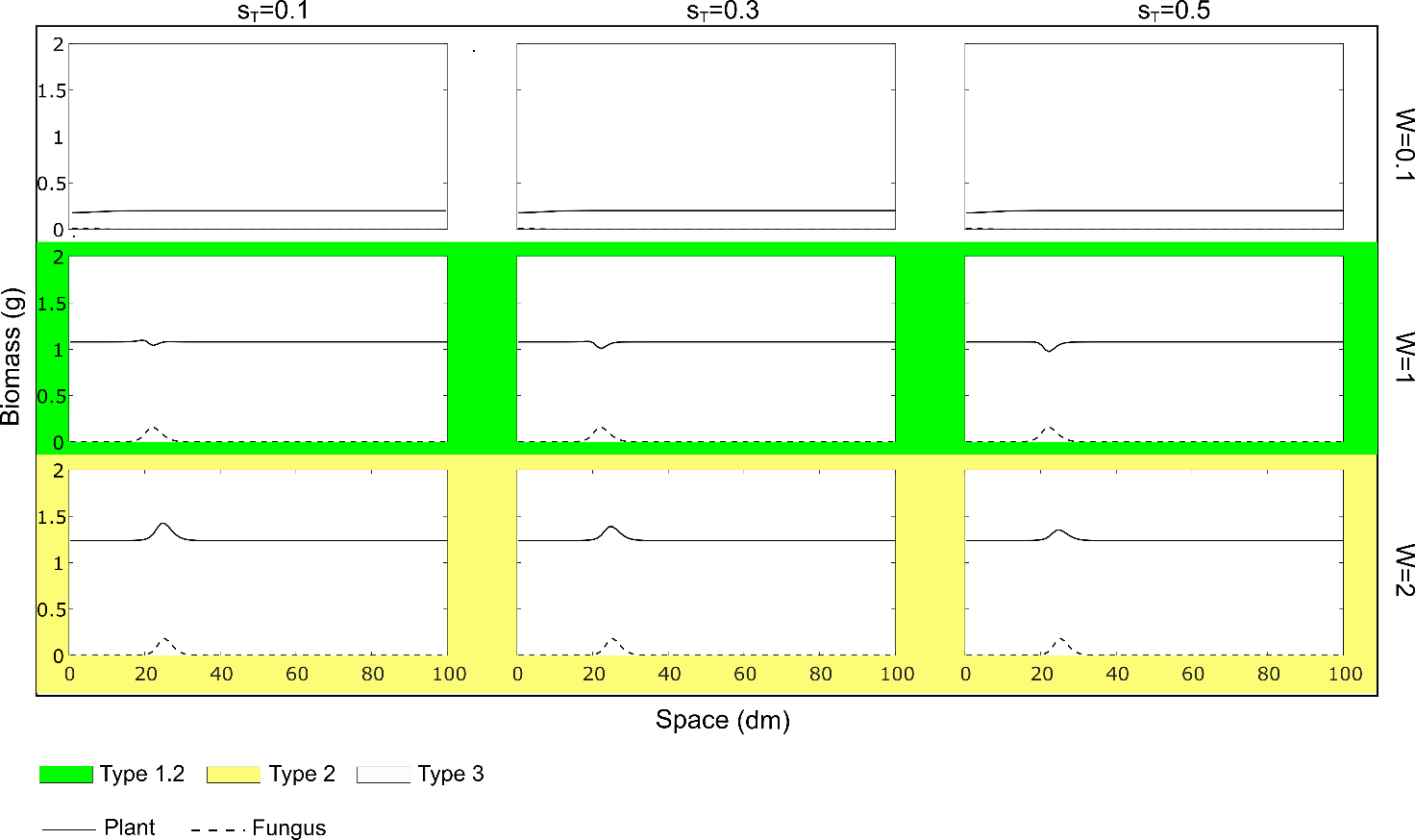
***

***Figure S5.*** *Detailed panel showing the results of the modelled plants and fungi biomasses (transects in space) for the combined hypotheses with g_F_ = 0.01. The parameters related to the levels of water inputs (W) and the accumulation of phytotoxicity (*$c_{T}$*) have been varied.*

*
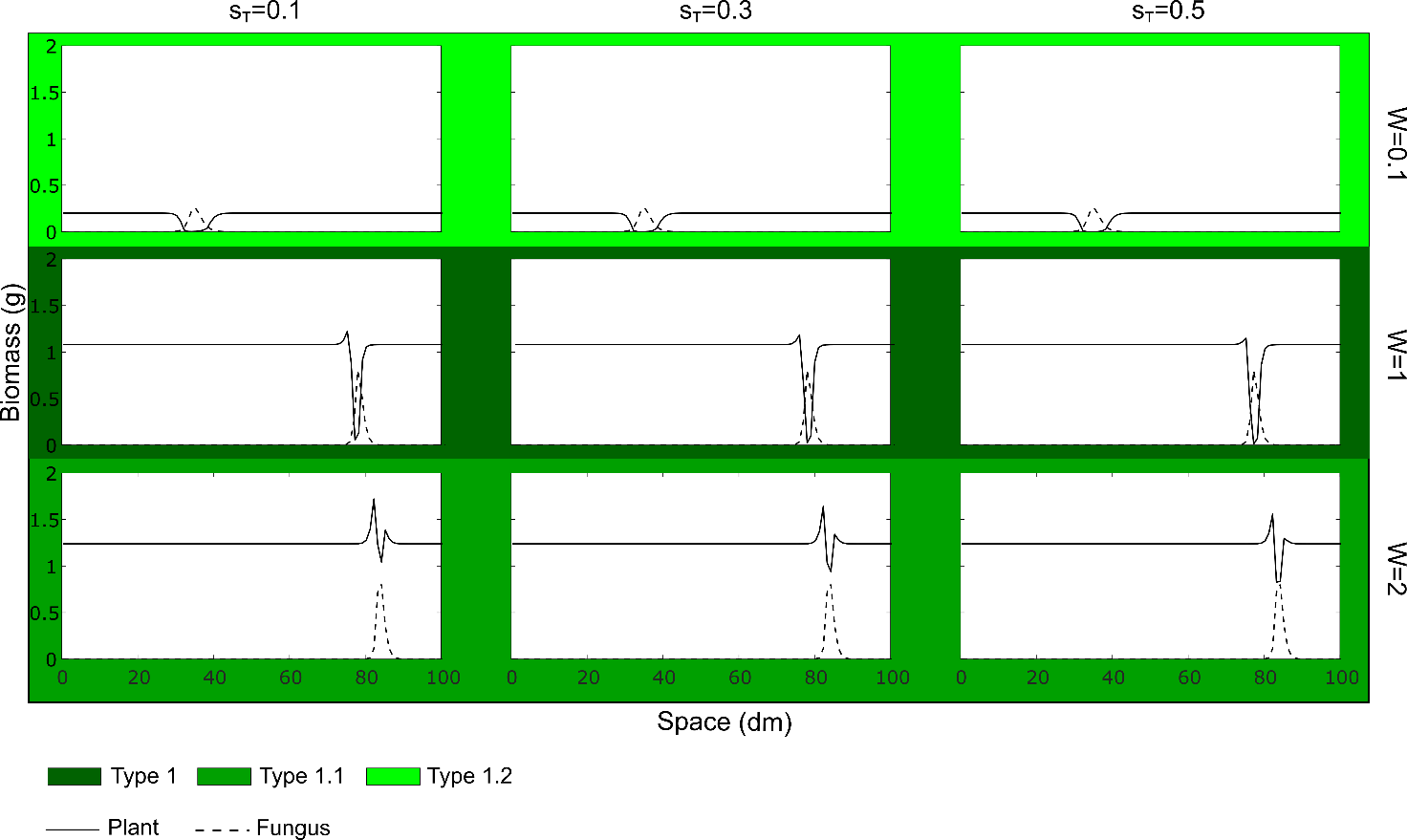
*

***Figure S6.*** *Detailed panel showing the results of the modelled plants and fungi biomasses (transects in space) for the combined hypothesis with g_F_ = 0.05. The parameters related to the levels of water inputs (W) and the accumulation of phytotoxicity (*$c_{T}$*) have been varied.*

*
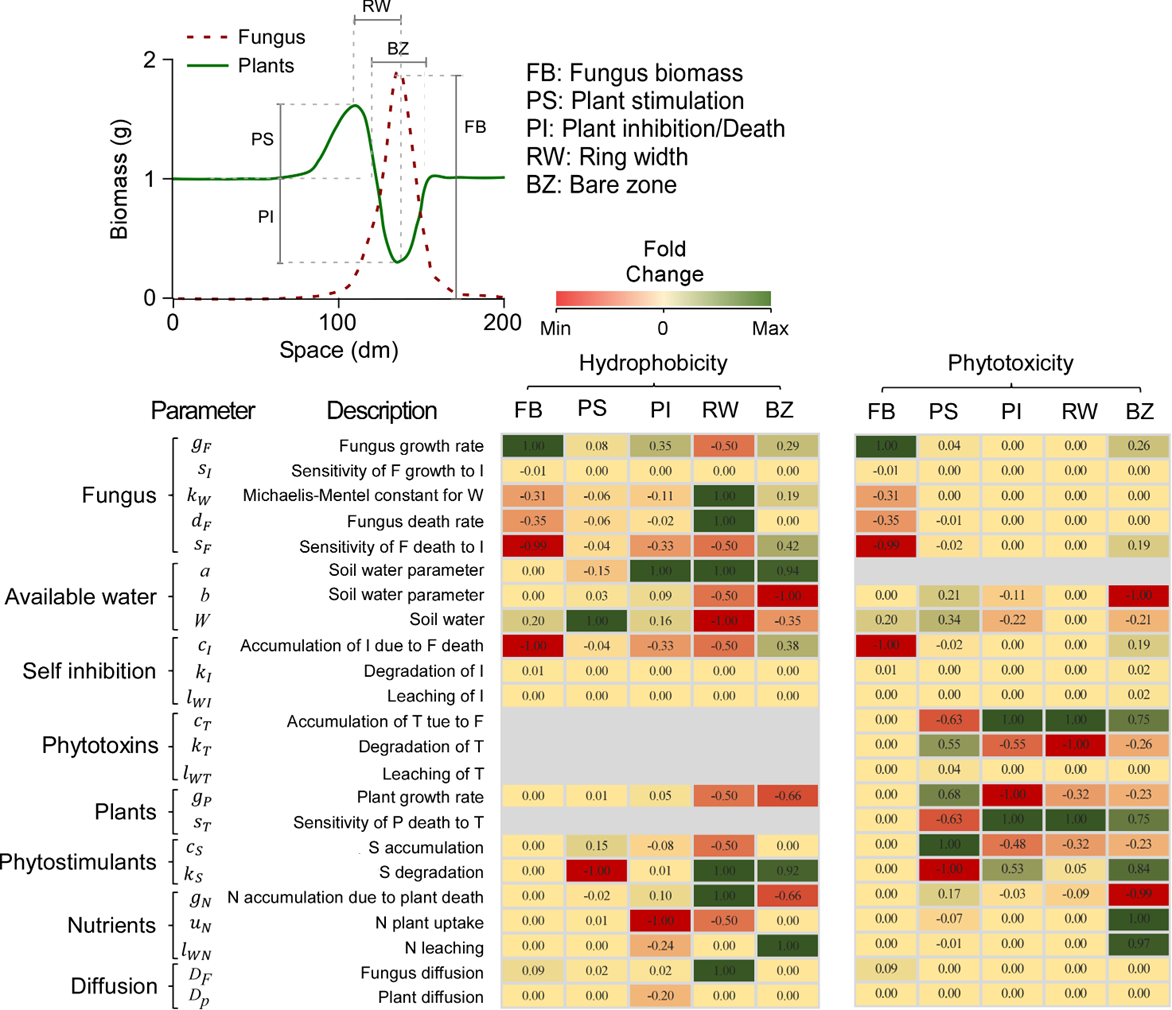
*

***Figure S7****. Heatmaps for the hydrophobicity and phytotoxicity hypotheses. The intensity of each colour is normalized per column. The grey rows show the parameters set to 0 for the corresponding hypothesis.*

***Video S1****. Spatial and temporal evolution of the model with the hydrophobicity as only negative effect on vegetation. Horizontal axis indicates space (dm), vertical axis indicates biomass (g) of both fungi (dashed line) and plants (solid line). Parameter values as reported in Table 1 except for W=1 and g_F_=0.05*

***Code S1****. Model code. The model is implemented in MATLAB 2020 (the MathWorks).*
